# Pulmonary hemosiderosis in children with Down syndrome: a national experience

**DOI:** 10.1186/s13023-018-0806-6

**Published:** 2018-04-20

**Authors:** Aurelia Alimi, Jessica Taytard, Rola Abou Taam, Véronique Houdouin, Aude Forgeron, Marc Lubrano Lavadera, Pierrick Cros, Isabelle Gibertini, Jocelyne Derelle, Antoine Deschildre, Caroline Thumerelle, Ralph Epaud, Philippe Reix, Michael Fayon, Sylvie Roullaud, Françoise Troussier, Marie-Catherine Renoux, Jacques de Blic, Sophie Leyronnas, Guillaume Thouvenin, Caroline Perisson, Aimé Ravel, Annick Clement, Harriet Corvol, Nadia Nathan, for the French RespiRare® group

**Affiliations:** 1 Assistance Publique Hôpitaux de Paris (APHP), Pediatric Pulmonology department and Reference centre for rare lung diseases, RespiRare, Trousseau Hospital, 75012 Paris, France; 2 APHP, Pediatric Pulmonology department, RespiRare, Necker Enfants Malades Hospital , 75015 Paris, France; 3 APHP, Pediatric Pulmonology department, RespiRare, Faculty Paris Diderot VII, Inserm U1149, Robert Debré Hospital, Paris, France; 4 Pediatric department, Hospital Center, Inserm U646, 72037 Le Mans, France; 5 Pediatric Pulmonology department, University Hospital, Rouen, France; 6 Pediatric Pulmonology department, University Hospital, Inserm 1078, Brest, France; 7 Pediatric department, University Hospital, Tours, France; 8 Pediatric department, University Hospital, Nancy, France; 9 Pediatric Pulmonology department, University Hospital, UMR CNRS 8204 - Inserm U1019, Lille, France; 10 Pediatric Pulmonology department, RespiRare, Créteil University Hospital, Inserm U955, Créteil, France; 11 Pediatric Pulmonology department, University Hospital, UMR CNRS 5558, Lyon, France; 12 Pediatric Pulmonology department, University Hospital, U1219, Bordeaux, France; 13 Pediatric department, Hospital Centre, Angouleme, France; 14 Pediatric Pulmonology department, University Hospital, Inserm U892, Angers, France; 15 Pediatric Pulmonology department, University Hospital, Inserm U1046, Montpellier, France; 16 Sorbonne Université, Inserm UMR-S938, Paris, France; 17 Institut Jérôme Lejeune, Paris, France; 18 Sorbonne Université, Inserm UMR-S933, Paris, France

**Keywords:** Pulmonary hemosiderosis, Down syndrome, Children, Autoimmunity, Interstitial lung disease, Celiac disease, Vasculitis, Pulmonary hypertension

## Abstract

**Background:**

Pulmonary hemosiderosis is a rare and complex disease in children. A previous study from the French RespiRare® network led to two important findings: 20% of the children presented with both pulmonary hemosiderosis and Down syndrome (DS), and at least one tested autoantibody was found positive in 50%. This study investigates the relationships between pulmonary hemosiderosis and DS.

**Methods:**

Patients younger than 20 years old and followed for pulmonary hemosiderosis were retrieved from the RespiRare® database. Clinical, biological, functional, and radiological findings were collected, and DS and non-DS patients’ data were compared.

**Results:**

A total of 34 patients (22 girls and 12 boys) were included, among whom nine (26%) presented with DS. The mean age at diagnosis was 4.1 ± 3.27 years old for non-DS and 2.9 ± 3.45 years old for DS patients. DS patients tended to present a more severe form of the disease with an earlier onset, more dyspnoea at diagnosis, more frequent secondary pulmonary hypertension, and an increased risk of fatal evolution.

**Conclusions:**

DS patients have a higher risk of developing pulmonary hemosiderosis, and the disease seems to be more severe in this population. This could be due to the combination of an abnormal lung capillary bed with fragile vessels, a higher susceptibility to autoimmune lesions, and a higher risk of evolution toward pulmonary hypertension. A better screening for pulmonary hemosiderosis and a better prevention of hypoxia in DS paediatric patients may prevent a severe evolution of the disease.

**Electronic supplementary material:**

The online version of this article (10.1186/s13023-018-0806-6) contains supplementary material, which is available to authorized users.

## Background

Pulmonary hemosiderosis is a rare lung disease characterised by the triad hemoptysis, iron deficiency anaemia, alveolar and/or interstitial opacities on lung imaging. Bronchoalveolar lavage (BAL) and/or lung biopsy ascertain the diagnosis. The BAL fluid is bloody with a hemosiderin-laden macrophage ratio above 30% and/or a Golde score higher than 50 [1]. Its frequency is poorly documented, but some authors hypothesised an incidence of 0.24–1.23 per million [2]. In children, very few cases are described worldwide. An association with celiac disease (Lane-Hamilton syndrome) and cow milk protein intolerance (Heiner syndrome) has been reported [3–6]. However, apart from the disease-specific condition, the aetiology and the pathophysiology of pulmonary hemosiderosis remain unknown. It is a chronic disease that commonly evolves in successive relapses separated by periods of remission. The prognosis is highly variable from a unique exacerbation with a complete recovery to multiple relapses with a risk of evolution toward lung fibrosis and terminal respiratory insufficiency. Corticosteroids are the mainstay of the treatment, with some children receiving also immunosuppressive drugs [7]. The French reference centre for rare lung diseases network RespiRare® previously reported a paediatric study population of 25 children aged 0.8 to 14 years old at diagnosis [8]. Two important findings were observed: 5 (20%) children presented with both pulmonary hemosiderosis and Down syndrome (DS), a percentage higher than expected; and at least one tested autoantibody was found positive in 50%. The present study aimed to investigate the newly documented relationships between pulmonary hemosiderosis and DS in the RespiRare® network.

## Materials and methods

### Patients

Prevalent patients with pulmonary hemosiderosis were retrieved from the national RespiRare® database with a query on the words pulmonary hemosiderosis, alveolar hemorrhage, siderophage, Golde score and hemoptysis. The database and data collection have been approved by French national data protection authorities (CNIL n°908,324 and CCTIRS n°08.015bis). Each patient and/or his or her legal representatives were informed prior to entering their data in the database. The charts of all the patients meeting the keywords were reviewed. Patients with a proven pulmonary hemosiderosis on BAL and/or lung biopsy between 1997 and 2017 were selected. Patients older than 20 years old at the time of the study were excluded.

### Data

The following data were collected from the RespiRare® database and analysed: age at diagnosis; gender; DS status; familial history of pulmonary hemosiderosis and/or autoimmune disease; initial symptoms; biological parameters, including hemoglobin (Hb), reticulocytes, autoantibodies (antinuclear antibodies [ANA], anti-cytoplasmic antibodies [ANCA], anti-smooth muscle, anti-cyclic citrullinated peptide [CCP], anti-proteinase-3 [PR-3], anti-myeloperoxidase [MPO], anti-DNA, anti-endomysium, anti-transglutaminase, anti-SSA, anti-cardiolipin) and rheumatoid factor (RF); lung imaging results, including chest X-ray and thoracic high-resolution computed tomography (HRCT); pulmonary function tests (PFT); BAL and histological results; type and durations of treatments; and evolution of the disease with a specific attention to the presence or absence of pulmonary arterial hypertension (PAH) and relapses. Relapses were defined by the presence of a hemoptysis and/or a respiratory exacerbation (defined according to the ChiLD criteria) [9] associated with either new radiological findings compatible with an alveolar bleeding or increased anaemia or deglobulisation.

### Statistics

The data from the pulmonary hemosiderosis patients with no DS, the non-DS group, were compared to those from the patients with both pulmonary hemosiderosis and DS, the DS group. Quantitative values were reported as median and range or mean and standard deviations. Qualitative data were reported as number (percentages). Comparisons between groups were established using a non-parametric *t*-test. *P* values less than 0.05 were considered statistically significant.

## Results

### Population clinical characteristics

**Fig. 1:**
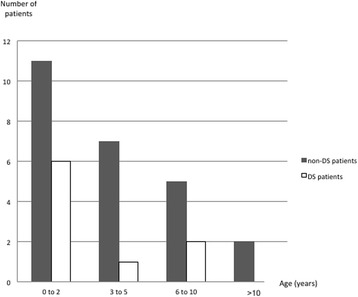
Age at presentation of the 34 included patients. Black bars represent the ages at presentation of the non-DS patients, and white bars represent the ages at presentation of the DS patients

A total of 42 paediatric patients were followed for pulmonary hemosiderosis in the RespiRare network. Eight patients were excluded because they were older than 20 years. The main clinical characteristics of the 34 included patients are presented in Table 1 and Additional file 1: Table S1. The age at diagnosis ranged from 3 days to 11.5 years old (Fig. 1). Among the 34 included patients, 9 (26%) presented with DS genetically confirmed (DS group); 8 had a free and homogeneous trisomy, and one patient had a partial trisomy with unbalanced translocation inherited from a balanced translocation in her mother.

**Table 1:** Main characteristics of the patients with pulmonary hemosiderosis

	All patients *n* = 34	Non-DS group *n* = 25	DS group *n* = 9	*P*-value
	n (%)	n (%)	n (%)	
Girls	22 (65%)	18 (72%)	4 (44%)	0.22
Boys	12 (35%)	7 (28%)	5 (56%)	0.22
Mean age at the diagnosis	3.80 ± 3.30	4.15 ± 3.27	2.92 ± 3.45	0.47
Family history				
Pulmonary hemosiderosis	3 (9%)	3(12%)	0 (0%)	0.55
Autoimmune disorder	2 (6%)	0 (0%)	2 (22%)	0.06
Personal history				
PAH	3 (9%)	0 (0%)	3 (33%)	0.01
Cardiopathy	4 (12%)	0 (0%)	4 (44%)	0.003
Symptoms at presentation				
Hemoptysis	16 (47%)	14 (56%)	2 (22%)	0.13
Cough	10 (29%)	8 (32%)	2 (22%)	0.69
Dyspnoea	23 (68%)	15 (60%)	9 (100%)	0.03
Pneumonia	9 (26%)	6 (24%)	3 (33%)	0.67
Minimal hemoglobin				
< 7 g/dl	20 (62.5%)	14* (61%)	6 (56%)	1
≥ 7 g/dl	12 (37.5%)	9* (39%)	3 (44%)	1

*Abbreviations: DS* down syndrome, *PAH* pulmonary arterial hypertension

*missing data for 2 patients

In the non-DS group (*n* = 25), the sex ratio was in favour of girls (72%). Three patients had a familial form of the disease (one had an uncle with pulmonary hemosiderosis and 2 were siblings), 2 had a biologically confirmed cow’s milk allergy (one diagnosed at the same time as the pulmonary hemosiderosis and the other 5 years before), and one patient was also diagnosed with type B Niemann Pick disease. In this group, none of the patients presented with cardiac comorbidities.

In the DS group (*n* = 9), the sex ratio was in favour of boys (56%). Two had a familial history of autoimmunity without pulmonary hemosiderosis, and 4 had a congenital cardiopathy. Two had identified PAH prior to the pulmonary hemosiderosis diagnosis. The other comorbidities are listed in Additional file 1: Table S1.

At diagnosis, dyspnoea was the most frequent symptom (*n* = 23, 68%). Hemoptysis was documented in only 16 (47%) of the patients (Table 1). The patients of the DS group presented with a higher frequency of dyspnoea (100% in the DS group vs. 60% in the non-DS group, *P* = 0.04) and less hemoptysis (22% vs. 56%, respectively; *P* = 0.1). Cough and pneumonia were also frequently reported at diagnosis in both groups.

### Investigations at diagnosis

**Fig. 2:**
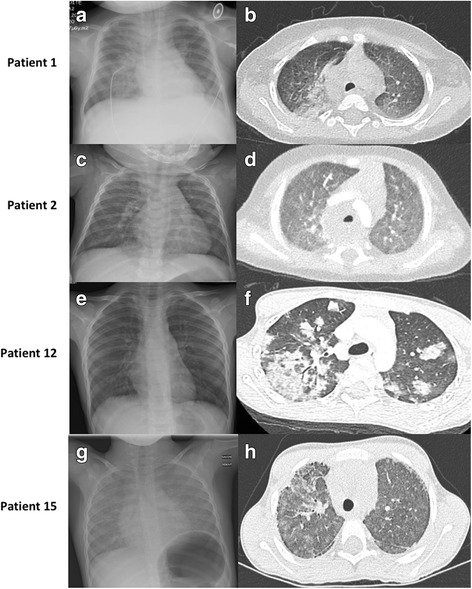
Lung imaging of two patients in the DS group (patients 1 and 2), and two in the non-DS group (patients 12 and 15). Panels **a** and **b** Chest x-ray and thoracic HRCT-scan of patient 1 at diagnosis (8 months of age) show bilateral alveolar opacities with a posterior predominance and diffuse ground glass opacities. Panels **c** and **d** Chest x-ray and thoracic HRCT-scan of patient two at 1 month of age show bilateral diffuse ground glass opacities. Panels **e** and **f** Chest x-ray and thoracic HRCT-scan of patient 12 at 4.3 years old show bilateral alveolar condensations with a patchy repartition, central and peripheral, and surrounding ground glass opacifications. Panels **g** and **h** Chest x-ray and thoracic HRCT-scan of patient 15 at 5 years old show bilateral patchy ground glass opacifications and signs of lung fibrosis with reticulations and sub-pleural cysts

In both groups, most patients presented with a severe anaemia (Hb < 7 g/dl). All the patients presented with an alveolar and interstitial pattern with a diffuse distribution of the lesions on the chest radiography and/or the HRCT scan (available for 28 [82%] patients, Fig. 2). The main abnormalities were ground-glass opacities, nodules, and alveolar condensations. Lung fibrosis was already present at the first evaluation for 2 patients (one in each group).

All BAL were consistent with the diagnosis of pulmonary hemosiderosis, with a median of 83% of hemosiderin-laden macrophages and/or a median Golde score of 168. Four patients (all in the non-DS group) underwent an open lung biopsy with a positive Perls’ staining for all.

At diagnosis, only 13 of the 34 patients were able to perform PFT because of their young age or their general or respiratory condition. PFT were normal in 7 (54%) patients and showed a restrictive, an obstructive, or a mixed syndrome in, respectively, 2 (15%), 1 (8%), and 2 (15%) patients. Diffusion capacity of the lung for carbon monoxide (DLCO) was measured in 4 patients and was below expected values (< 75%) for 3 of them, 2 and 1 in the non-DS group and the DS group, respectively.

**Fig. 3:**
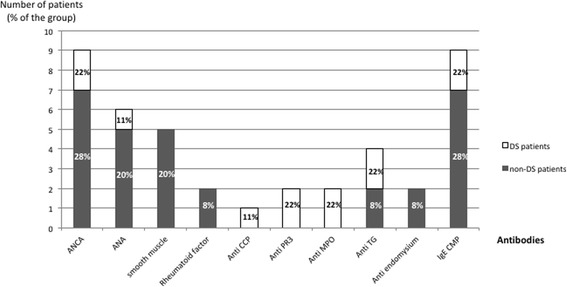
Number of patients with positive antibodies in each group. The black bars represent the number of non-DS patients with positive antibodies and the white bars represent the number of DS patients with positive antibodies. The % in each bar is the % of patients in each group with positive antibodies. Abbreviations: DS = Down syndrome, ANCA = anti-cytoplasmic antibodies; ANA = antinuclear antibodies; CCP = anti-cyclic citrullinated peptide; PR3 = anti-proteinase 3; MPO = anti-myeloperoxidase; TG = anti-transglutaminase; DS = Down syndrome

As previously described, a large number of the patients presented with biological signs of autoimmunity (Table 2): 24 (75%) patients had at least one positive antibody, 18 (78%) in the non-DS group and 6 (67%) in the DS group. In both groups, ANA were the most frequently observed antibodies (*n* = 11, 32%); the other positive antibodies were ANCA, anti-smooth muscle, RF, anti-CCP, anti-PR-3, anti-MPO, anti-DNA, anti-endomysium, anti-transglutaminase, anti-SSA and anti-cardiolipin. Their repartition between both groups is listed in Fig. 3.

**Table 2:** Positive antibodies at diagnosis in the pulmonary hemosiderosis cohort

Positive antibodies	Total (32 patients)*	non-DS group (23 patients)*	DS group (9 patients)	*P*-value
	n (%)	n (%)	n (%)	
1	12 (34%)	9 (39%)	3 (33%)	0.78
2	7 (19%)	6 (26%)	1 (11%)	0.64
> 2	5 (12%)	3 (13%)	2 (22%)	0.60
Total	24 (75%)	18 (78%)	6 (67%)	0.04

*Abbreviations:* *DS* down syndrome

*Missing data for 2 patients in the non-DS group

### Treatment

Treatment information was available for 32 patients. All the patients received systemic corticosteroids as first-line treatment. Monthly intravenous pulses of methylprednisolone (300 mg/m^2^/day for 3 days) were used for four (12%) patients, oral corticosteroids (1 to 2 mg/kg/day) for 9 (28%) patients, and both for 19 (59%) patients. A long-term treatment was necessary for the majority of them, with 30% of the patients still receiving corticosteroids after 1 year of follow-up, and 25% after 5 years. Because the three deceased patients belonged to the DS group, the duration of the corticosteroid treatment could not be compared between both groups.

More than 25% of the patients were treated with second-line therapies. A total of ten patients received hydroxychloroquine (31%): nine patients in the non-DS group and one in the DS group. Immunosuppressive drugs (mycophenolate mofetil, cyclophosphamide and/or azathioprine) were used for nine patients (28%): six (26%) in the non-DS group and three (33%) in the DS group. Beside drugs, patients with cow’s milk protein allergies were treated with an exclusion diet [10].

### Evolution

The mean length of follow-up was 4.9 [0.8–18.3] years; most of the patients (*n* = 25, 73.5%) were followed for more than 3 years. For 13 (40.6%) patients, pulmonary hemosiderosis occurred only as a single event, with no relapse after treatment (Table 3). Nineteen patients (59.3%) experienced at least one relapse: 13 (56.5%) patients in the non-DS group and six (67%) in the DS group (*P* = 0.63). Five patients, all in the DS group, presented with a PAH. The PAH pre-existed the pulmonary hemosiderosis for two and was secondary for three patients. Three of them died after multiple relapses from acute PAH and/or massive pulmonary hemorrhage at age 0.7, 2 and 7 years old, respectively. For these three patients, the pulmonary hemosiderosis was diagnosed within the first months of life.

**Table 3:** Evolution of the patients with pulmonary hemosiderosis

	Total (32 patients)*	non-DS group (23 patients)*	DS group (9 patients)	*P*-value
	n (%)	n (%)	n (%)	
No relapses	13 (40.6%)	10 (43.4%)	3 (33.3%)	0.7
Relapses	19 (59.3%)	13 (56.5%)	6 (67%)	0.46
Pulmonary arterial hypertension	5 (14.7%)	0	5 (55%)	0.0003
Death	3 (9%)	0	3 (33%)	0.0003

*Abbreviations:* *DS* down syndrome

*Missing data for 2 patients in the non-DS group

## Discussion

Pulmonary hemosiderosis is a very rare disease in children, and its pathophysiology remains unclear. We report here our national experience through the RespiRare® network. This study highlights the surprising over-representation of DS in pulmonary hemosiderosis paediatric patients. DS is the most common genetic disorder, with a prevalence reaching 140 per 100,000 children [11]. Therefore, in our pulmonary hemosiderosis cohort population of 34 patients, 0 to 1 patient with DS was expected. However, nine children, i.e., around a quarter of the pulmonary hemosiderosis population, presented with DS. Pulmonary hemosiderosis in DS patients has not been reported on so far, except through isolated case reports [12, 13]. Moreover, based on our national findings, the estimated prevalence of pulmonary hemosiderosis in children reaches 1.85 per 1,000,000 children, compared to 138.5 per 1,000,000 DS children.

Patients with and without DS displayed remarkable differences. In the DS group, six patients out of 9 were younger than 3 years old at diagnosis, whereas in the non-DS patients, two-thirds of the patients were older than 3 years old [8, 14–16]. Although hemoptysis is a classic sign of the disease, it was present in fewer than half of the patients, whereas dyspnoea was the most frequent respiratory symptom. DS patients seemed to present a more severe form of the disease with an earlier onset, more dyspnoea at diagnosis, more secondary PAH and a major risk of fatal evolution.

In this study, autoimmunity stigma was documented in a large majority of the patients in both groups (75%, *n* = 24). The link between pulmonary hemosiderosis and the presence of circulating autoantibodies is not clearly understood in patients with no valid diagnosis criteria for vasculitis [8]. DS, particularly in men, is known to be associated with a high incidence of autoimmune disorders such as thyroiditis, hypothyroidism, type 1 diabetes, Addison disease, celiac disease, and other, rarer, disorders, including primary sclerosing cholangitis [17]. Lungs are not considered a privileged target for DS autoimmunity, but autoantibodies are frequently found in DS patients even with no evidence of clinical autoimmune disease [18]. Recent studies reported the crucial role in DS autoimmune dysfunction of the autoimmune regulator protein (AIRE) located on chromosome 21. *AIRE* is selectively expressed in the thymus and is a transcription factor for many tissue-restricted antigens that enhance the generation of regulatory T-cells and consecutively induce a central tolerance. It is presumed to protect against autoimmune diseases. Bi-allelic mutations of *AIRE* are associated with an autoimmune disease that is similar to the spectrum of autoimmunity observed in DS [19]. In DS, despite three expressed copies of *AIRE*, the overall *AIRE* expression was shown to be reduced compared to controls. All together, these findings favour a central role of *AIRE* in DS autoimmune disorders [20, 21]. Autoimmunity could be one of the links between DS and pulmonary hemosiderosis. In our study, several antibodies were found exclusively in DS or non-DS patients, but the majority of the patients had positive circulating antibodies, with no significant differences between groups. Surprisingly, DS patients did not receive more immunosuppressive agents than those of the non-DS group (*P* = 0.41). The reasons for fewer prescriptions of immunosuppressive drugs in DS patients are unclear. A hypothesis could be that clinicians were avoiding the risk of major sensitivity to chemotherapy in DS patients [22–24].

Another hypothesis to explain the association between pulmonary hemosiderosis and DS could be an altered alveolar and vascular development of the lungs. It is known that children with DS have more microscopic pulmonary malformations and present an increased risk for PAH development, independently from cardiac malformations [25]. Histological descriptions have shown elements in favour of arrested lung development such as alveolar simplification, persistence of a double capillary network, prominence of a bronchial circulation or, more recently, intrapulmonary bronchopulmonary anastomoses [26]. Lung epithelial development is closely related to signalling from the vascular compartment: an inhibition of the vascular endothelial growth factor (VEGF) induces an altered angiogenesis and an abnormal alveolar structure development in the foetus [27]. Several anti-angiogenic factors are located on chromosome 21: endostatin (*COL18A1*), beta-amyloid protein (*APP*), and regulator of calcineurin 1 (*RCAN1*). These factors are overexpressed during the DS foetal period due to the three copies of the genes. It has recently been shown that their up-regulation in DS lung tissues was associated with a reduced vessel density and an increase of the vessel wall thickness compared to non-DS lung tissues [28]. The in-utero capillary development of the DS foetus is consistent with the hypothesis of an altered maturation of the capillary network of the alveoli and the absence of regression of the thick arterial musculature of the pulmonary vessels [29]. This impaired vascular development could be responsible for an altered alveolar maturation with simplified large alveoli. The reduced total alveolar surface associated with an abnormal capillary network could constitute a risk factor for hypoxemia, PAH and alveolar hemorrhage.

Patients with DS have additional risk factors for PAH due to chronic hypoxia and recurrent hypoxic events such as frequent congenital heart diseases, lung infections, recurrent aspirations, and obstructive sleep apnea syndrome (OSAS) [30]. OSAS is observed in up to half of adult DS patients [31]. In children, extreme prevalence between 0 and 100% has been reported in small cohorts [30, 32, 33]. Multiple factors can explain OSAS in children with DS, but the main causes include hypotonia, facial dysmorphia with macroglossia and narrow upper airways. It has also been suggested that tonsillar growth in the first months of life could increase the airway collapse [34]. Central apnoea reported in DS patients can also increase the OSAS severity. Untreated OSAS increases chronic hypoxia and, subsequently, PAH development. Altogether, in DS patients, the severity of pulmonary hemosiderosis could be due to the combination of a higher susceptibility to autoimmune lesions of the alveolar capillary, an abnormal lung capillary bed and a higher PAH risk. These pathophysiologic hypotheses could shed further new light on possible abnormal lung maturation in non-DS patients with pulmonary hemosiderosis.

## Conclusion

This study reports for the first time a higher risk of severe pulmonary hemosiderosis in DS paediatric patients. Because alveolar bleeding symptoms can be inconspicuous, it could be suggested to perform a chest X-ray in all DS patients with chronic unexplained anaemia and/or chronic, unexplained dyspnoea. At this stage, only hypotheses can be proposed on the links between DS and pulmonary hemosiderosis such as an increased risk of PAH. If such a hypothesis is confirmed by further studies, systematic sleep investigations in DS patients could be proposed to screen for OSAS and to prevent PAH. For all pulmonary hemosiderosis patients, with or without DS, autoimmune explorations are critical at diagnosis and may be repeated regularly. In the era of genomic research, DS patients’ aggregation in such a rare disease could be a real opportunity to link chromosome 21 genes to new pathophysiologic clues for pulmonary hemosiderosis.

## Additional file

Additional file 1: **Table S1.** Detailed characteristics of the 34 included patients. (DOCX 24 kb)

## Abbreviations

AIRE: Autoimmune regulator protein
ANA: Antinuclear antibodies
ANCA: Anti-cytoplasmic antibodies
APP: Beta-amyloid protein
BAL: Bronchoalveolar lavage
CCP: Cyclic citrullinated peptide
COL18A1: Gene encoding the endostatin
CS: Corticosteroids
DLCO: Diffusion capacity of the lung for carbon monoxide
DS: Down syndrome
F: Female
Hb: Hemoglobin
HCQ: Hydroxychloroquine
HRCT: High-resolution computed tomography
M: Male
MD: Missing data
MMF: Mycophenolate mofetil
MPO: Myeloperoxidase
OSAS: Obstructive sleep apnea syndrome
PAH: Pulmonary arterial hypertension
PFT: Pulmonary function tests
PR3: Proteinase 3
RCAN1: Regulator of calcineurin 1
RF: Rheumatoid factor
TG: Transglutaminase
VEGF: Vascular endothelial growth factor
