# Supplementary material for: Pulmonary hemosiderosis in children with Down syndrome: a national experience
Source: Orphanet J Rare Dis. 2018 Apr 20;13:60. doi: 10.1186/s13023-018-0806-6 (PMC5910623; doi:10.1186/s13023-018-0806-6)
Supplement: Supplementary file 1 — Table S1. Detailed characteristics of the 34 included patients. (DOCX 24 kb) [file 13023_2018_806_MOESM1_ESM.docx]

**Additional file for**

**Hemosiderosis in children with Down syndrome: a national experience**

Aurelia Alimi^1^, Jessica Taytard^1^, Rola Abou Taam^2^, Véronique Houdouin^3^, Aude Forgeron^4^, Marc Lubrano Lavadera^5^, Pierrick Cros^6^, Isabelle Gibertini^7^, Jocelyne Derelle^8^, Antoine Deschildre^9^, Caroline Thumerelle^9^, Ralph Epaud^10^, Philippe Reix^11^, Michael Fayon^12^, Sylvie Roullaud^13^, Françoise Troussier^14^, Marie-Catherine Renoux^15^, Jacques de Blic^2^, Sophie Leyronnas^1^, Guillaume Thouvenin^1,16^, Caroline Perisson^1^, Aimé Ravel^17^, Annick Clement^1,18^, Harriet Corvol^1,16^, Nadia Nathan^1,18^, for the French RespiRare® group

| **Table S1: Detailed characteristics of the 34 included patients** | | | | | | | | | | |
| --- | --- | --- | --- | --- | --- | --- | --- | --- | --- | --- |
| **Patient's number** | **non-DS / DS** | **Gender (M/F) / Age at onset (years)** | **Age at the study time (years)** | **Length of follow-up (years)** | **Initial presentation** | **Minimal Hb**  **(g/dl)** | **Positive antibodies** | **PAH** | **Treatments** | **Follow-up** |
| 1 | DS | M / 0.7 | 2 | 1.3 | Acute respiratory distress with PAH and cardiac arrest with no hemoptysis | 5.3 | CCP, ANCA, MPO, PR3 | Yes | 5 IV CS pulses + oral CS | No relapse |
| 2 | DS | F / 0 | Died at 8 months | 0.8 | Congenital cardiopathy. Dyspnoea and hemoptysis shortly after birth | 11.3 | Cow milk proteins | Yes | 3 IV CS pulses + oral CS | Deceased from PAH and terminal respiratory insufficiency |
| 3 | DS | F / 9.1 | 12.2 | 13.8 | Acute dyspnoea and cough | 4.2 | No | Yes | Oral CS | No relapse |
| 4 | DS | M / 0.4 | Died at age 2 | 1.7 | Cough, dyspnoea, hemoptysis | 6.3 | ANA, PR3, MPO, ANCA | No | Oral CS | 5 relapses. Deceased at age 2 from severe alveolar hemorrhage |
| 5 | DS | F / 4.5 | 19.6 | 15.1 | Balanced translocation t(10;21)(p12.1;q21)  Acute respiratory distress | 3.2 | No | No | IV CS pulses + oral CS + azathioprine | 4 relapses |
| 6 | DS | M / 2.6 | 13.4 | 6.1 | Congenital cardiopathy. Dyspnoea with fever | 3.9 | DNA | No | Oral CS + MMF | Many relapses |
| 7 | DS | M / 0 | Died at age 7.3 | 7.3 | Congenital cardiopathy. Spastic dyspnoea with fever, lung fibrosis | 9.1 | Transglutaminases | Yes | IV CS pulses, HCQ | PAH, many relapses |
| 8 | DS | F / 0.7 | 10.8 | 6.5 | Congenital cardiopathy. Autoimmune hepatitis, portal hypertension, sclerosing cholangitis. Acute respiratory distress with fever | 11.3 | No | Yes | Oral CS for 5 years | No relapse |
| 9 | DS | M / 6.1 | 8.0 | 1.3 | Hypothyroidism  Chronic anaemia | 12.3 | No | No | 20 IV CS pulses + oral CS + azathioprine | One relapse |
| 10 | Non-DS | F / 4.4 | 10.8 | 11,7 | Dyspnoea and hemoptysis | 11 | RF, Cow milk proteins, smooth muscle | No | IV CS pulses + oral CS + HCQ + MMF + cyclophosphamide | Multiple relapses with dyspnoea and hemoptysis |
| 11 | Non-DS | F / 2.4 | 9.6 | 2.6 | Dyspnoea and hemoptysis | 6.6 | RF, Cow milk proteins | No | IV CS pulses + oral CS | Multiple relapses with hemoptysis |
| 12 | Non-DS | F / 4.3 | 7.2 | 2.8 | Hemoptysis, chronic anaemia | 6.9 | ANA | No | IV CS pulses + oral CS + HCQ | Two relapses |
| 13 | Non-DS | M / 6.4 | 8.6 | 2.2 | Dyspnoea and hemoptysis with fever | 2.7 | Cow milk proteins | No | IV CS pulses + oral CS | One relapse after 2 years |
| 14 | Non-DS | F / 11.6 | 15.9 | 1.8 | Dyspnoea and hemoptysis | 11.7 | ANCA | No | One year oral CS | No relapse, lung fibrosis |
| 15 | Non-DS | F / 4.8 | 10.4 | 5.8 | Chronic anaemia, chronic asthma, lung fibrosis | 11.7 | No | No | IV CS pulses + oral CS | Chronic dyspnoea, restrictive syndrome with lung fibrosis. |
| 16 | Non-DS | F / 7.2 | 16.8 | 4.1 | Acute dyspnoea | 2.6 | Gliadin, transglutaminase, endomysium | No | Initial oral CS | No relapse after gluten eviction |
| 17 | Non-DS | F / 1.9 | 11.8 | 5.1 | Cough, hemoptysis, familial case | 5.3 | ANCA | No | 41 IV CS pulses | No relapse |
| 18 | Non-DS | M / 2.3 | 11.8 | 4.2 | Cough, no hemoptysis, familial case | 7.6 | ANCA | No | Initial 6 IV CS pulses + oral CS | No relapse |
| 19 | Non-DS | F / 1.1 | 21.1 | 18.3 | Acute respiratory distress and cardiac arrest | 2.3 | Smooth muscle | No | IV CS pulses + 15 years oral CS + 13 years HCQ | No relapse |
| 20 | Non-DS | F / 0.1 | 17.7 | 8 | Hemoptysis, dyspnoea | 9.6 | ANA, smooth muscle | No | 10 years IV CS pulses + oral CS | 5 relapses |
| 21 | Non-DS | F / 5.9 | 18.8 | 9.9 | Hemoptysis, dyspnoea | 3.8 | MD | No | 5 years oral CS + 8 years HCQ + MMF | Multiple relapses |
| 22 | Non-DS | F / 6.3 | 18.8 | 2.8 | Hemoptysis, dyspnoea | 3 | RF, DNA, gliadin, endomysium | No | 3 years oral CS + 3 years HCQ | One relapse |
| 23 | Non-DS | F / 5.1 | 16.8 | 6.2 | Acute dyspnoea and cough | 1.9 | ANA, DNA, RF, smooth muscle | No | Oral CS + HCQ + MMF | No relapse |
| 24 | Non-DS | F / 4.2 | 6.9 | 2 | Dyspnoea, no hemoptysis | 3.8 | ANCA | No | IV CS pulses + oral CS + azathioprine + cyclophosphamide | Multiple relapses |
| 25 | Non-DS | F / 5.3 | 8.8 | 2,3 | Hemoptysis, dyspnoea | 4 | ANA | No | IV CS pulses + oral CS + HCQ | 2 relapses (hemoptysis), lung fibrosis |
| 26 | Non-DS | F / 1.9 | 9.6 | MD | MD | MD | ANCA | No | MD | MD |
| 27 | Non-DS | F / 7.8 | 18.3 | 4.1 | Niemann Pick B  Chronic cough | 11.1 | No | No | IV CS pulses + oral CS | Dyspnoea on exertion |
| 28 | Non-DS | F / 0.2 | 7.5 | MD | MD | MD | MD | No | MD | MD |
| 29 | Non-DS | M / 1.4 | 12.6 | 6 | Dyspnoea, familial case | 5 | No | No | IV CS pulses + oral CS + HCQ + MMF | Many relapses, 8 months oxygen requirement |
| 30 | Non-DS | M / 0 | 6.5 | 1.3 | Hemoptysis and acute respiratory distress with cardiac arrest | 8.6 | ANCA | No | IV CS pulses | No relapse, no more treatment |
| 31 | Non-DS | M / 6.1 | 10.8 | 3.8 | Dyspnoea, chronic anaemia, no hemoptysis | 7.2 | Cow milk proteins | No | IV CS pulses + oral CS + HCQ + Azathioprine + Cyclophosphamide | One relapse. Oxygen requirement |
| 32 | Non-DS | M / 0.3 | 2.3 | MD | Hemoptysis and acute respiratory distress with cardiac arrest | 11.6 | Cow milk proteins, ANCA | No | 6 IV CS pulses | Chronic cough and spastic dyspnoea |
| 33 | Non-DS | M / 0.1 | 7.7 | 7.6 | Cough, dyspnoea, hemoptysis | 11.2 | Cow milk proteins, transglutaminase | No | One IV CS pulses + oral CS | No relapse after eviction of cow milk proteins |
| 34 | Non-DS | F / 10.6 | 14.5 | 8 | Cough, dyspnoea, hemoptysis | 13.5 | No | No | 5 IV CS pulses + oral CS | Multiple relapses. Stable for 4 years, no more treatment |

*Abbreviations*: Hb = hemoglobin; DS = Down syndrome; M = male; F = female; PAH = pulmonary hypertension; CCP= anti-cyclic citrullinated peptide; ANCA= anti-cytoplasmic antibodies; MPO = anti-myeloperoxidase antibody; PR3= anti-proteinase 3 antibody; ANA= antinuclear antibody; RF= rheumatoid factor; CS= corticosteroid; HCQ=hydroxychloroquine; MMF= mycophenolate mofetil; MD = missing data
